# Supplementary material for: Telomere-to-telomere assemblies of cattle and sheep Y-chromosomes uncover divergent structure and gene content
Source: Nat Commun. 2024 Sep 27;15:8277. doi: 10.1038/s41467-024-52384-5 (PMC11436988; doi:10.1038/s41467-024-52384-5)
Supplement: Supplementary file 1 — Supplementary Information [file 41467_2024_52384_MOESM1_ESM.pdf]

Telomere-to-telomere assemblies of Cattle and Sheep Y-chromosomes uncover divergent structure and gene content

Supplementary Information

Supplementary figures

Figure S1: Genome quality assessment with Merqury k-mer spectra plots
Figure S2: Whole genome assembly visualization with Bandage
Figure S3: Definition of the PAR boundaries: IGV sequence coverage and alignment visualization of the female haplotype long reads mapping
Figure S4: The loci of the ampliconic genes on the cattle (A) and the sheep (B) Y chromosomes
Figure S5: Cross-species phylogeny of the protein-coding ampliconic genes
Figure S6: Percentage identity of the centromeric HOR copies on the cattle Y-chromosome
Figure S7: The cattle Y chromosome-derived 73bp monomeric unit
Figure S8: The cattle Y-chromosome centromere
Figure S9: The sheep Y-chromosome centromere
Figure S10: Mashmap alignment between the BTAU5-Y (43.3Mbp) and the T2T cattle (59.4Mb)
Figure S11: Mashmap alignment dot plot between the Hu Sheep and the T2T sheep Y-chromosome (x-axis).

Supplementary methods

Supplementary Method 1: Defining the PAR boundaries
Supplementary Method 2: Repeat elements annotation
Supplementary Method 3: Satellite DNA annotation with Tandem Repeats Finder (TRF)
Supplementary Method 4: CENP-A enrichment analysis
Supplementary Method 5: Methylated Cytosines analysis
Supplementary Method 6: Transcript level quantification on the Y-chromosomes

SUPPLEMENTARY FIGURES

A

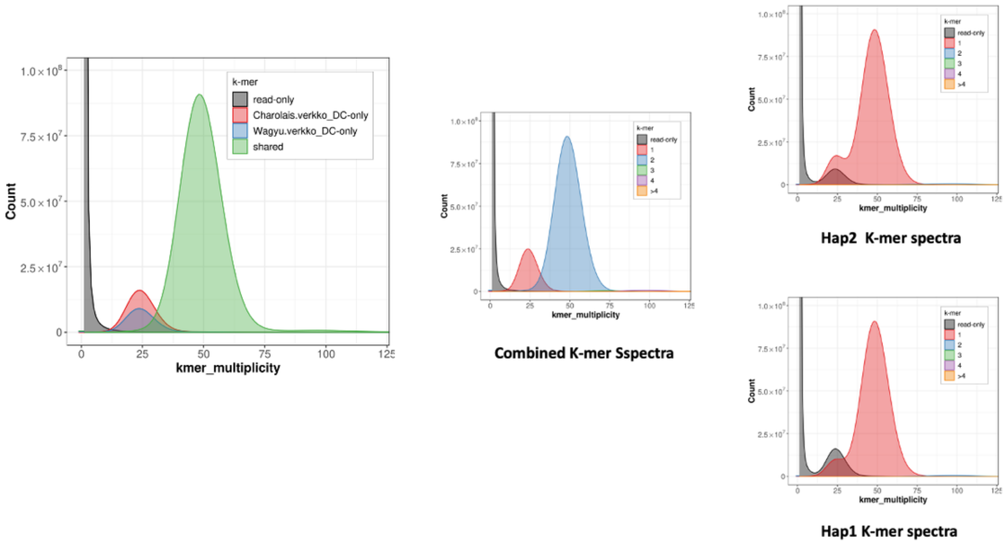

B

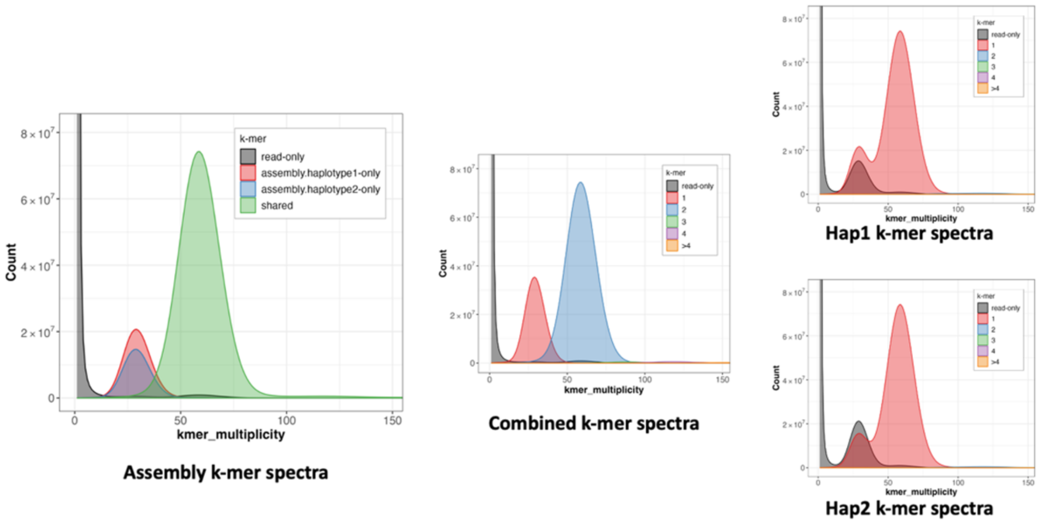

Figure S1: Genome quality assessment with Merqury k-mer spectra plots. Merqury[1] k-mer spectra plots of the genome assemblies of the F1 individuals from the (A) Wagyu_x_Charolais cattle and the (B) Churro_x_Friesian sheep crosses. The assembly k-mer spectra shows the distribution of the unique k-mers in the two haplotypes by highlighting the heterozygous regions (parental k-mers unique to either haplotype colored in red and blue) and the homozygous region (common to both haplotypes, colored in green). The survival of the reads k-mers in an assembly is an indication of the completeness of the assembly.

1. Whole genome assembly visualization with Bandage

(A) (B)

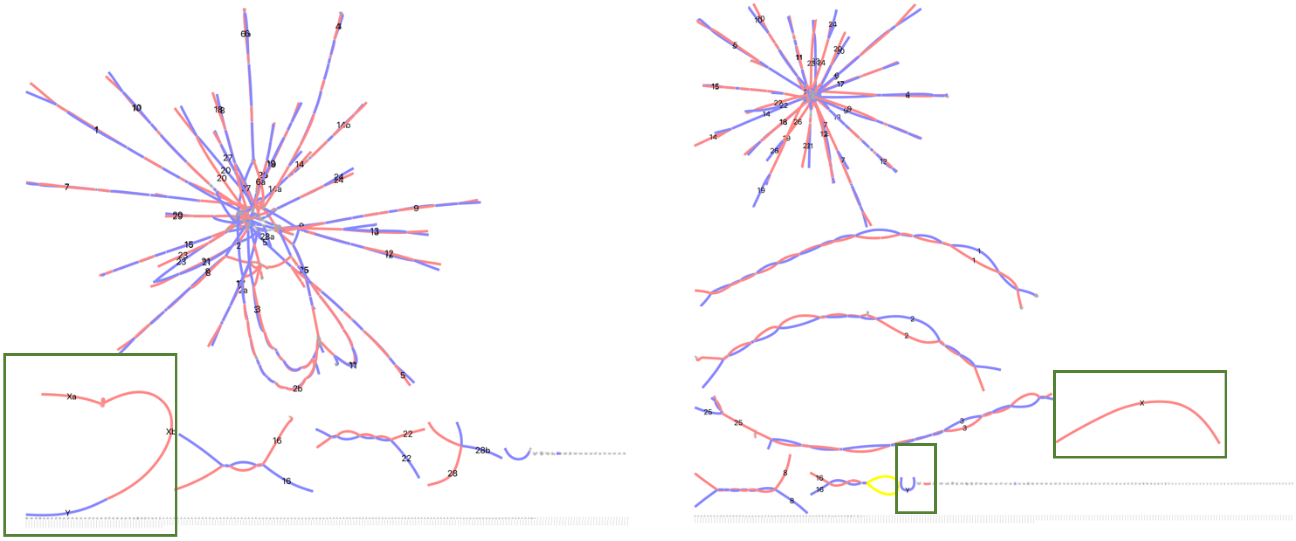

Figure S2: Whole genome assembly visualization with Bandage[2] showing the complete haplotype-resolved assemblies. The contigs of the paternal haplotypes are in blue color while those of the maternal haplotypes are colored in red. (A) For cattle, the sex chromosomes are highlighted in the green box showing a connection between the X- (red color in 2 contigs) and the Y-chromosome in a single contigs (blue) (B) The sheep sex chromosomes are both in single contigs and also highlighted in green boxes with the Y chromosome in blue color.

Pseudo-autosomal region (PAR) boundary definition

(A)

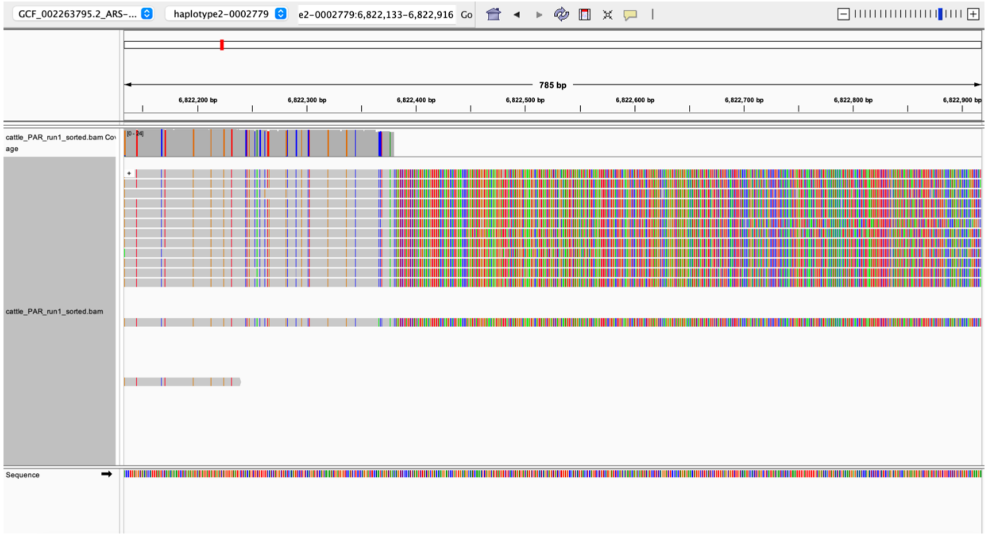

(B)

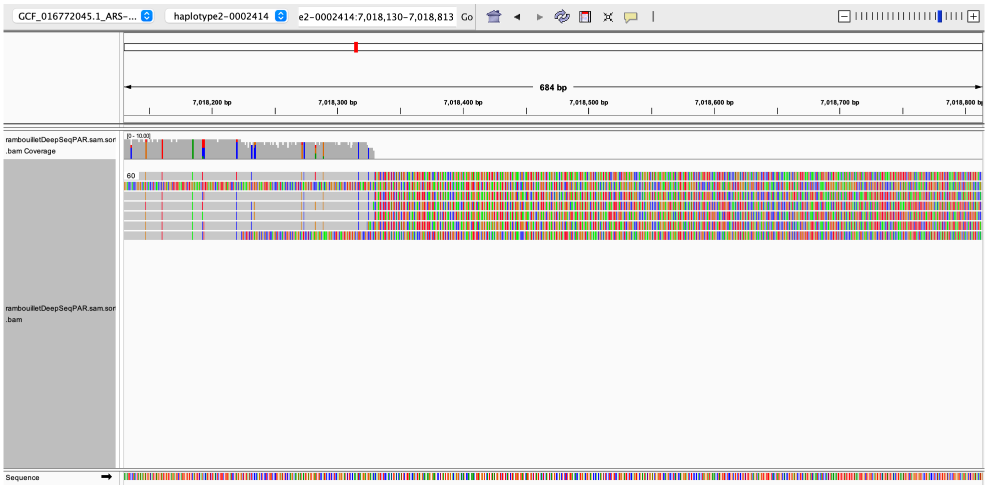

Figure S3: IGV[3] sequence coverage and alignment visualization of the female haplotype long reads mapping to (A) the cattle and (B) the sheep Y-chromosomes. The highly colored regions of the alignment track show 3’ end soft clipping of the HiFi reads at the PAR boundaries due to non-alignment of the sequences to the Y-chromosomes. Homology between the Y-chromosome and the X-chromosome is lost at the PAR boundaries where the Y chromosome transitions into the X-degenerate region. The coverage tracks above also show a complete drop of sequence coverage to zero at the PAR boundaries.

(A)

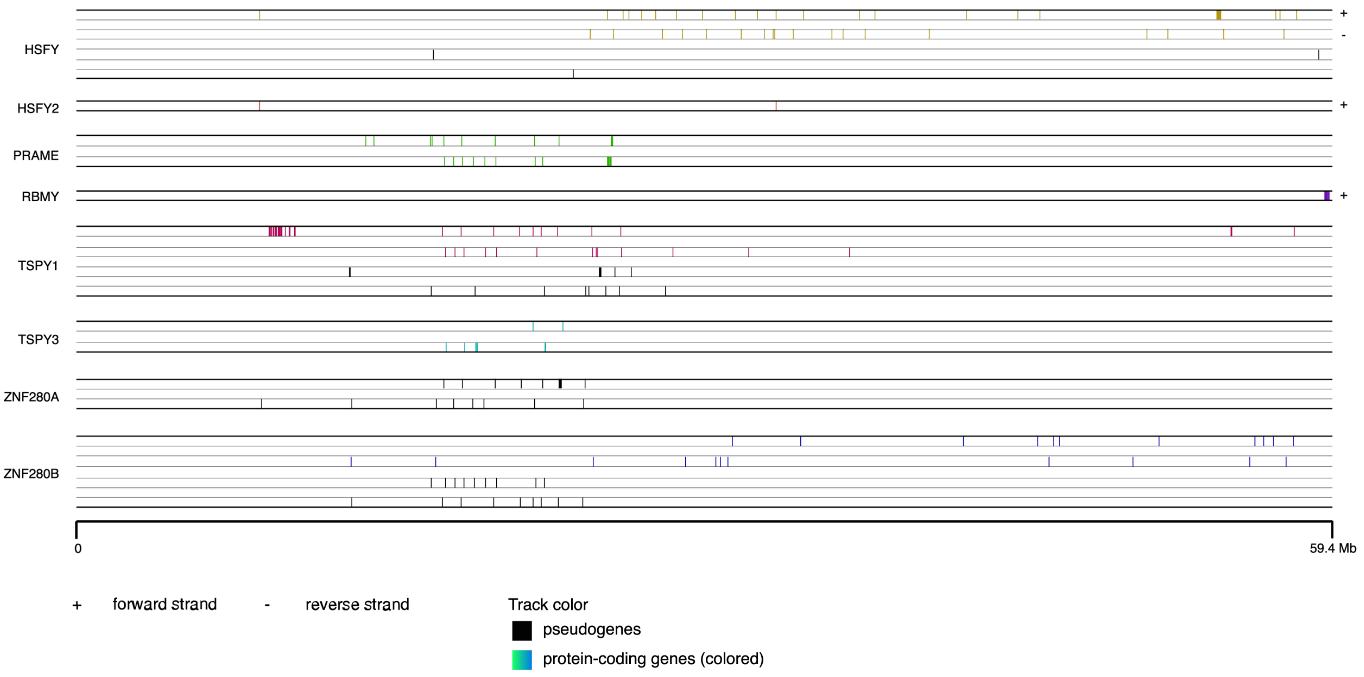

(B)

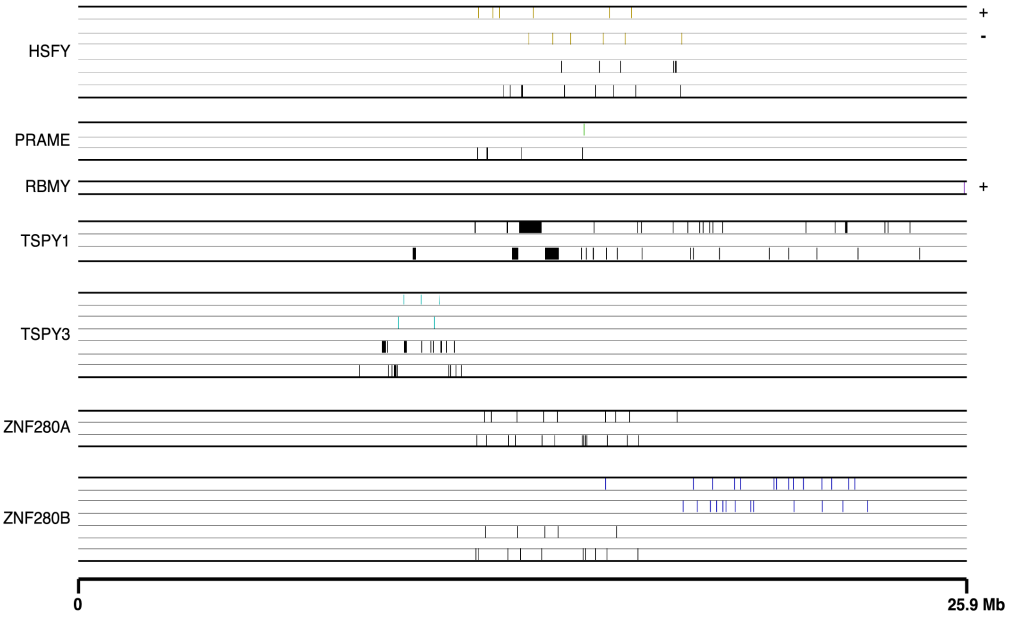

Figure S4: The loci of the ampliconic genes on the cattle (A) and the sheep (B) Y chromosomes rendered in four tracks for each gene; protein-coding genes (colored) on the positive and the negative strand are shown on tracks (from top) 1 and 2 respectively, while pseudogenes (black color) on the positive and negative strands are shown on tracks 3 and 4, respectively. Where there is only one track, its orientation is indicated with + or - sign. The *TSPY1* island is located

on the cattle-Y harboring a tandem array of 44 copies out of the total 68 protein-coding copies. Tandem arrays of protein-coding copies of *HSFY, PRAME, and RBMY* were also located on the Cattle-Y (thicker blocks on the tracks) but absent on the Sheep-Y. Both chromosomes did not have protein-coding copies of *ZNF280A*.

A

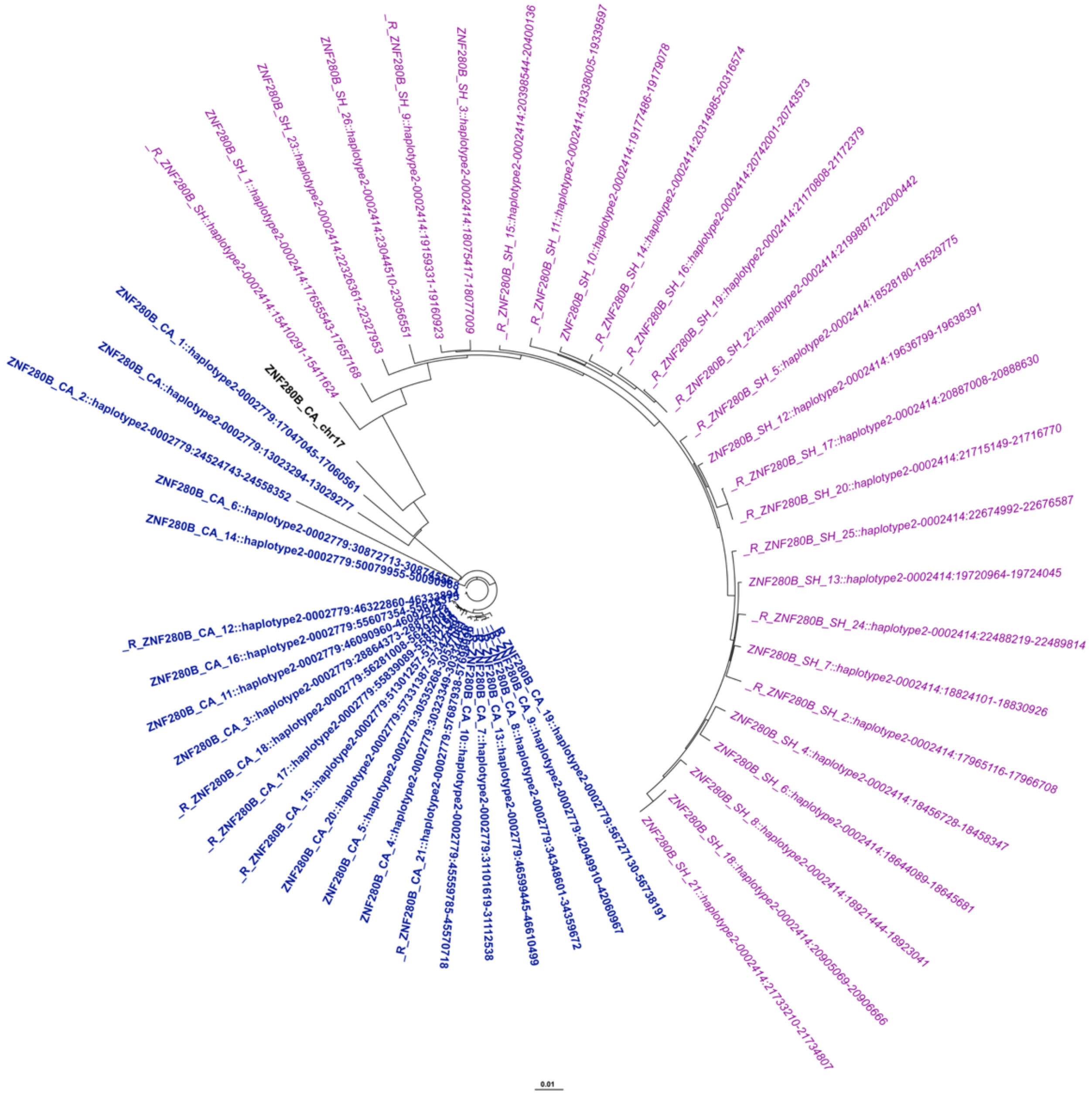
_R_ZNF280B_SH::haplotype2-0002414:15410291-15411624
ZNF280B_SH_1::haplotype2-0002414:17655543-17657168
ZNF280B_SH_23::haplotype2-0002414:22326361-22327953
ZNF280B_SH_26::haplotype2-0002414:23044510-23056551
_R_ZNF280B_SH_9::haplotype2-0002414:19159331-19160923
ZNF280B_SH_3::haplotype2-0002414:18075417-18077009
_R_ZNF280B_SH_15::haplotype2-0002414:20398544-20400136
_R_ZNF280B_SH_11::haplotype2-0002414:19338005-19339597
ZNF280B_SH_10::haplotype2-0002414:19177486-19179078
_R_ZNF280B_SH_14::haplotype2-0002414:20314985-20316574
_R_ZNF280B_SH_16::haplotype2-0002414:20742001-20743573
_R_ZNF280B_SH_19::haplotype2-0002414:21170808-21172379
_R_ZNF280B_SH_22::haplotype2-0002414:21998871-22000442
_R_ZNF280B_SH_5::haplotype2-0002414:18528180-18529775
_R_ZNF280B_SH_12::haplotype2-0002414:19636799-19638391
ZNF280B_SH_17::haplotype2-0002414:20887008-20888630
_R_ZNF280B_SH_20::haplotype2-0002414:21715149-21716770
_R_ZNF280B_SH_25::haplotype2-0002414:22674992-22676587
ZNF280B_SH_13::haplotype2-0002414:19720964-19724045
_R_ZNF280B_SH_24::haplotype2-0002414:22488219-22489814
ZNF280B_SH_7::haplotype2-0002414:18824101-18830926
_R_ZNF280B_SH_2::haplotype2-0002414:17965116-17966708
ZNF280B_SH_4::haplotype2-0002414:18456728-18458347
ZNF280B_SH_6::haplotype2-0002414:18644089-18645681
ZNF280B_SH_8::haplotype2-0002414:18921444-18923041
ZNF280B_SH_18::haplotype2-0002414:20905069-20906666
ZNF280B_SH_21::haplotype2-0002414:21733210-21734807
ZNF280B_CA_chr17
ZNF280B_CA_1::haplotype2-0002779:17047045-17060561
ZNF280B_CA::haplotype2-0002779:13023294-13029277
ZNF280B_CA_2::haplotype2-0002779:24524743-24558352
ZNF280B_CA_19::haplotype2-0002779:56727130-56738191
ZNF280B_CA_9::haplotype2-0002779:42049910-42060967
ZNF280B_CA_8::haplotype2-0002779:34348601-34359672
ZNF280B_CA_13::haplotype2-0002779:46599445-46610499
0.01

B

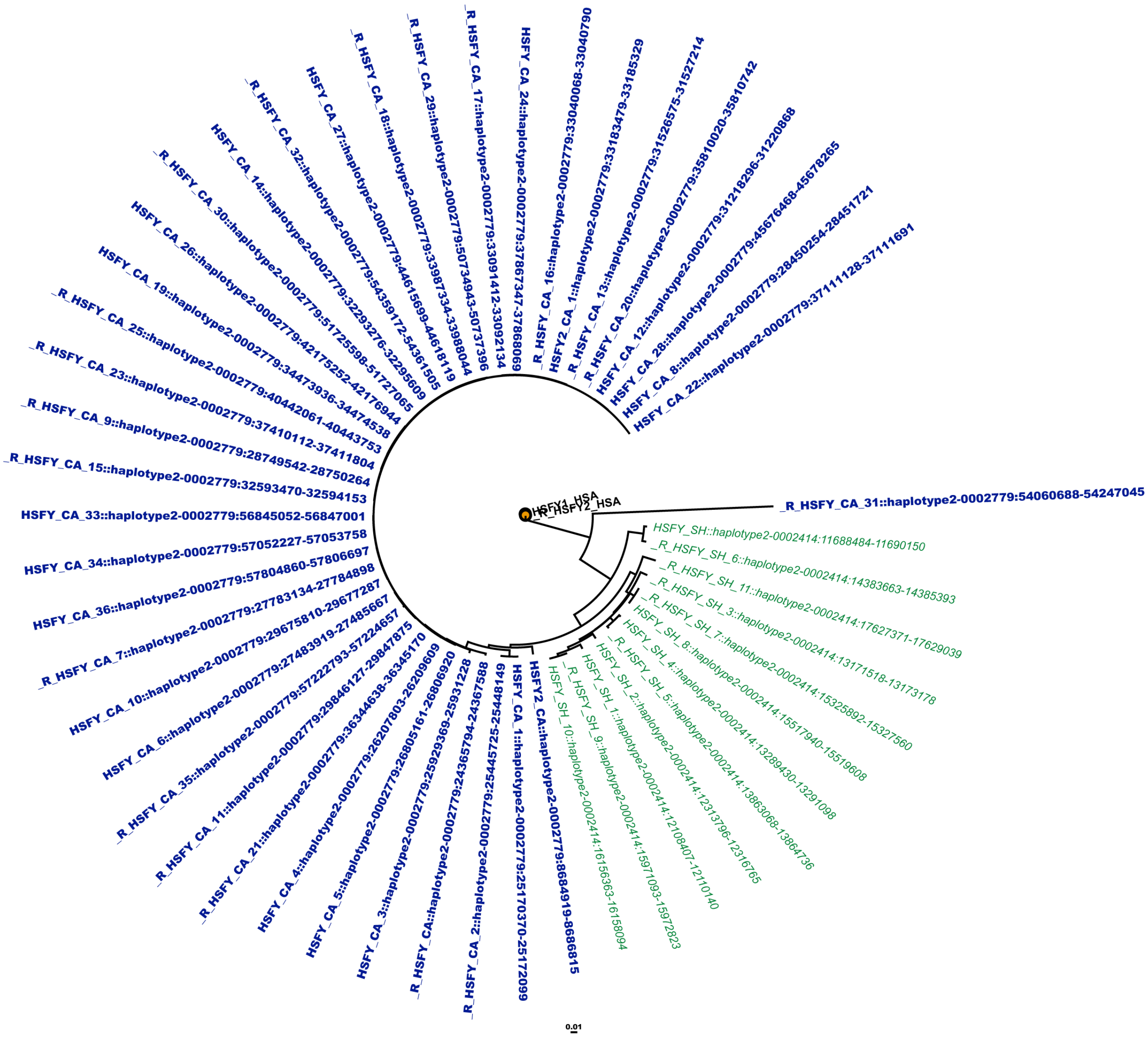

R_HSFY_CA_31::haplotype2-0002779:54060688-54247045
HSFY1_HSA
R_HSFY2_HSA
HSFY_SH::haplotype2-0002414:11688484-11690150
R_HSFY_SH_6::haplotype2-0002414:14383663-14385393
R_HSFY_SH_11::haplotype2-0002414:17627371-17629039
R_HSFY_SH_3::haplotype2-0002414:13171518-13173178
R_HSFY_SH_7::haplotype2-0002414:15325892-15327560
R_HSFY_SH_8::haplotype2-0002414:15517940-15519608
HSFY_SH_4::haplotype2-0002414:13289430-13291098
HSFY_SH_5::haplotype2-0002414:13863068-13864736
R_HSFY_SH_2::haplotype2-0002414:12313796-12316765
HSFY_SH_1::haplotype2-0002414:12108407-12110140
R_HSFY_SH_9::haplotype2-0002414:15971093-15972823
HSFY_SH_10::haplotype2-0002414:16156363-16158094
HSFY2_CA::haplotype2-0002779:8684919-8686815
HSFY_CA_1::haplotype2-0002779:25170370-25172099
R_HSFY_CA_2::haplotype2-0002779:25445725-25448149
R_HSFY_CA::haplotype2-0002779:24365794-24367588
HSFY_CA_3::haplotype2-0002779:25929369-25931228
HSFY_CA_5::haplotype2-0002779:26805161-26806920
HSFY_CA_4::haplotype2-0002779:26207803-26209609
R_HSFY_CA_21::haplotype2-0002779:36344638-36345170
R_HSFY_CA_11::haplotype2-0002779:29846127-29847875
R_HSFY_CA_35::haplotype2-0002779:57222793-57224657
HSFY_CA_6::haplotype2-0002779:27483919-27485667
HSFY_CA_10::haplotype2-0002779:29675810-29677287
R_HSFY_CA_7::haplotype2-0002779:27783134-27784898
HSFY_CA_36::haplotype2-0002779:57804860-57806697
HSFY_CA_34::haplotype2-0002779:57052227-57053758
HSFY_CA_33::haplotype2-0002779:56845052-56847001
R_HSFY_CA_15::haplotype2-0002779:32593470-32594153
R_HSFY_CA_9::haplotype2-0002779:28749542-28750264
R_HSFY_CA_23::haplotype2-0002779:37410112-37411804
R_HSFY_CA_25::haplotype2-0002779:40442061-40443753
HSFY_CA_19::haplotype2-0002779:34473936-34474538
HSFY_CA_26::haplotype2-0002779:42175252-42176944
R_HSFY_CA_30::haplotype2-0002779:51725598-51727065
HSFY_CA_14::haplotype2-0002779:32293276-32295609
R_HSFY_CA_32::haplotype2-0002779:54359172-54361505
HSFY_CA_27::haplotype2-0002779:44615699-44618119
R_HSFY_CA_18::haplotype2-0002779:33987334-33988044
R_HSFY_CA_29::haplotype2-0002779:50734943-50737396
R_HSFY_CA_17::haplotype2-0002779:33091412-33092134
HSFY_CA_24::haplotype2-0002779:37867347-37868069
R_HSFY_CA_16::haplotype2-0002779:33040068-33040790
HSFY2_CA_1::haplotype2-0002779:33183479-33185329
R_HSFY_CA_13::haplotype2-0002779:31526575-31527214
R_HSFY_CA_20::haplotype2-0002779:35810020-35810742
HSFY_CA_12::haplotype2-0002779:31218296-31220868
HSFY_CA_28::haplotype2-0002779:45676468-45678265
HSFY_CA_8::haplotype2-0002779:28450254-28451721
HSFY_CA_22::haplotype2-0002779:37111128-37111691
0.01

C

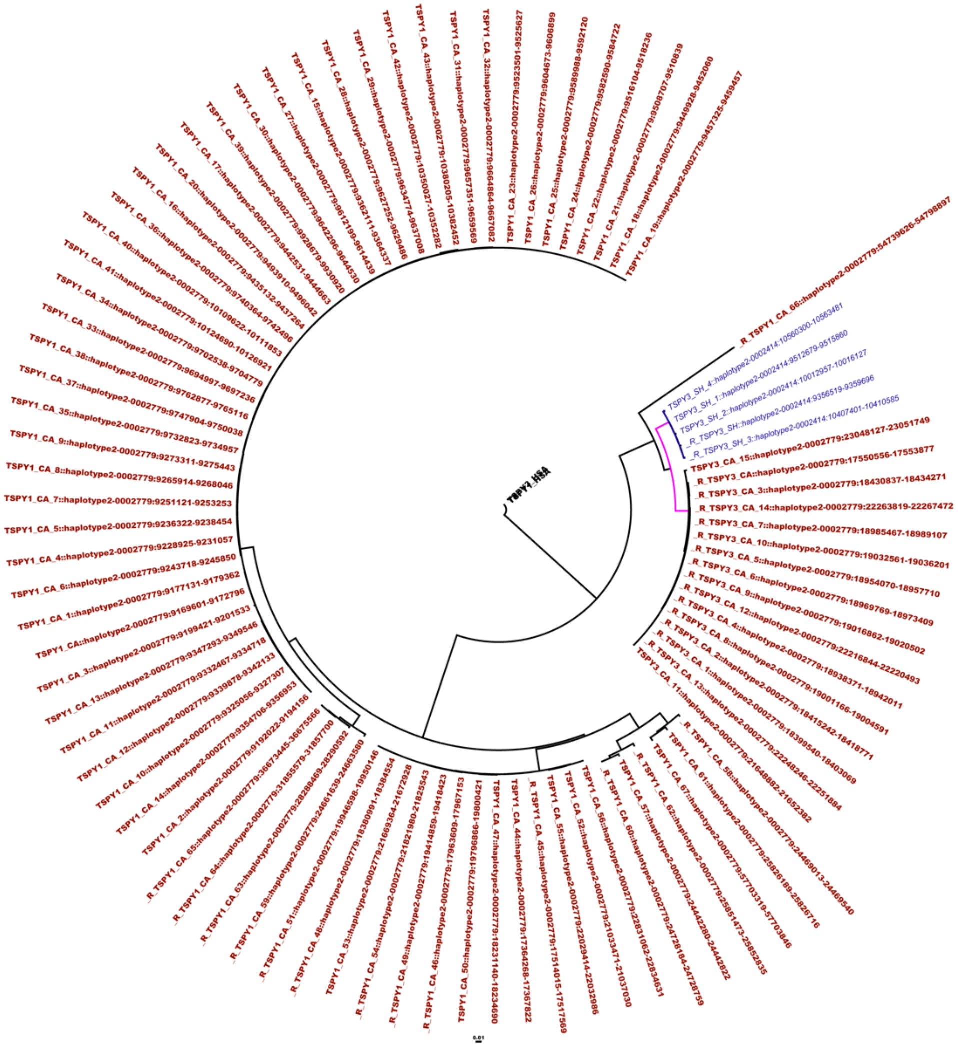

Figure S5: Inter-species clustering of the copies of the protein-coding ampliconic genes *HSFY* (A) *ZNF280B* (B) HSFY and (C) *TSPY* on the cattle and the sheep Y-chromosomes showing clear differentiation of the sheep copies from the cattle copies. *RBMY* and *PRAME* are not shown since only single copies were located on the sheep Y-chromosome. The genes in black font are the human copies used as outgroup for the tree construction. Since *ZNF280B* is bovine-specific, the copy in black font was obtained from cattle chromosome 17 from where it translocated to the Y-chromosome before amplification[4]. The sheep copies are in italicised normal font and have _SH appended to the gene name while the cattle copies are in bold with _CA appended to the gene name. The numbers between the name and the symbol "::" indicate the position of the copy on the chromosome relative to the end of the p-arm. The sheep copies are highligted in different colors for each gene family - *ZNF280B* (purple), *HSFY* (green), and *TSPY* (blue). The pink branch on panel (C) are all copies of *TSPY3* while the rest of the copies are *TSPY1*; the *TSPY3* copies were clearly separated from the *TSPY1* and were differentiated between cattle and sheep.

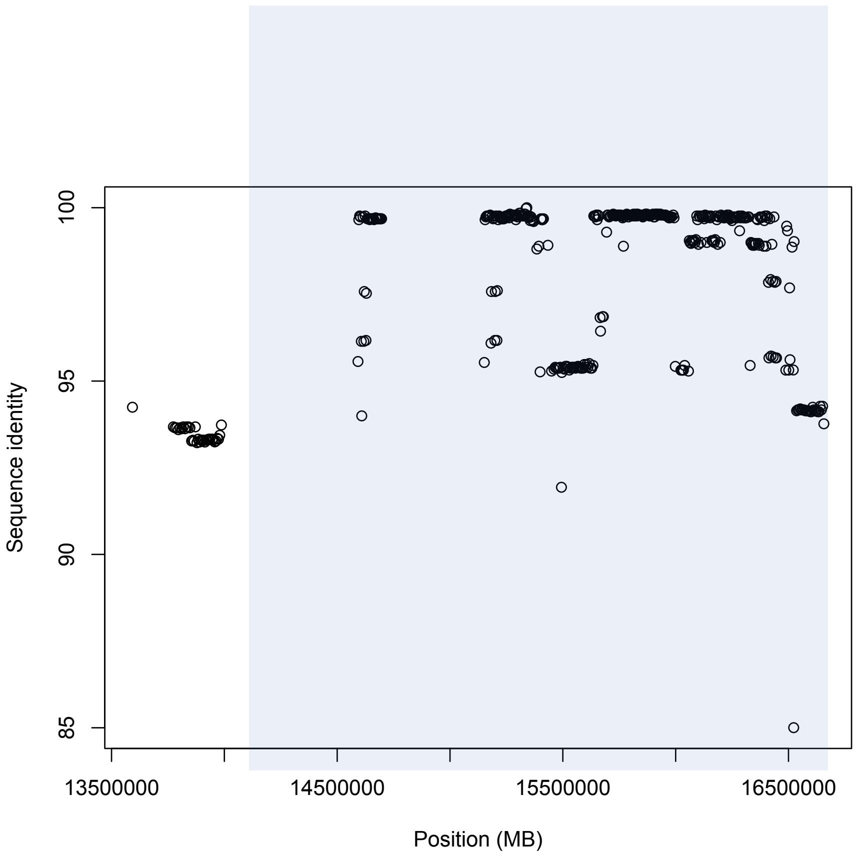

Figure S6: Percentage identity of the centromeric HOR copies on the cattle Y-chromosome. Sequence identity plot of the centromeric HOR repeat unit at the cattle centromere. The centromeric region is highlighted in light blue color. The HOR units flanking the centromere have lower sequence identity than the copies within the centromere. (Source data are provided as a Source Data file).

A

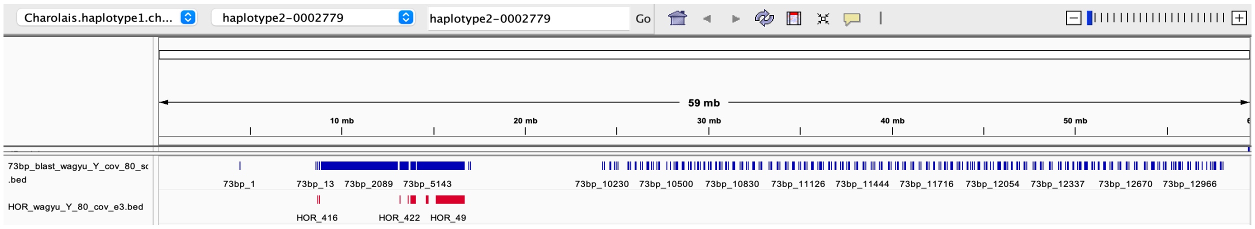

B

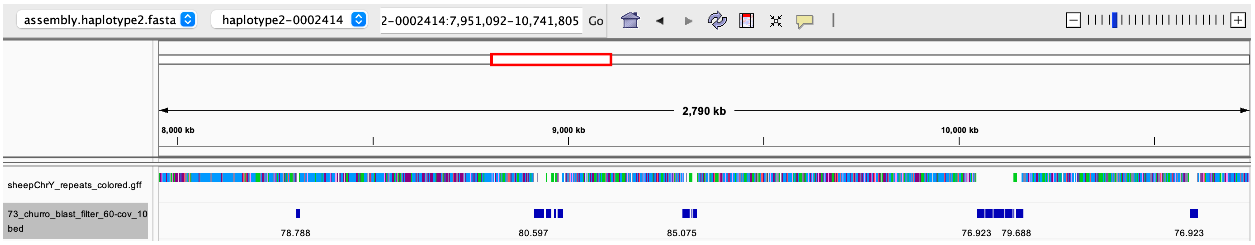

Figure S7: (A) The cattle Y chromosome-derived 73bp monomeric unit (blue track) spanning the length of the cattle Y-chromosome but enriched at the centromere, and the 3.7kb higher order repeat (HOR) (red track) also enriched at the centromere. (B) The 73bp monomeric unit on the sheep (second track) showing few diverged copies which are not proximal to the centromere locus.

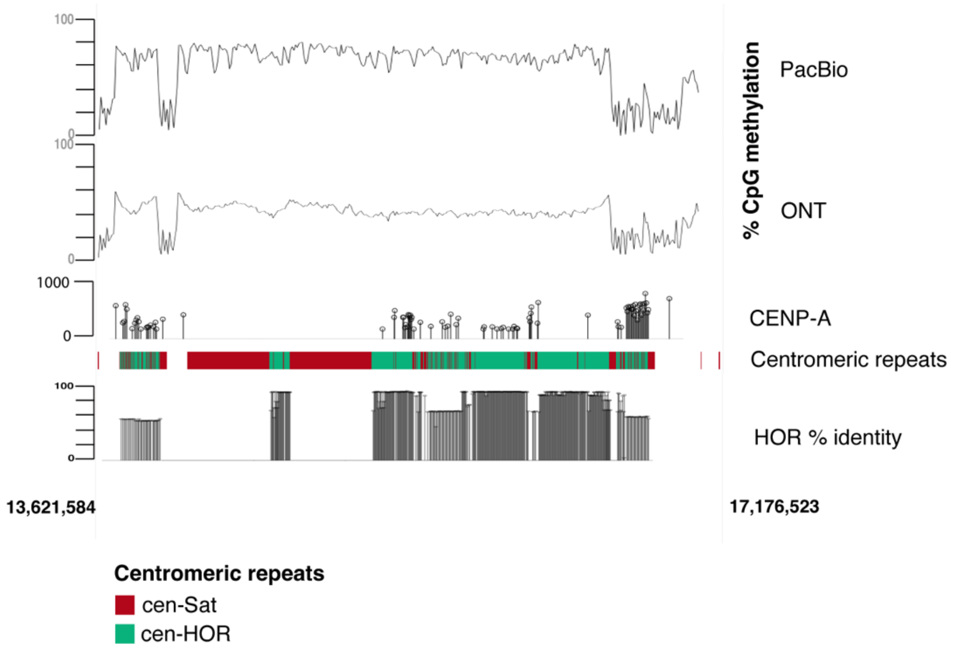

Figure S8: The cattle Y-chromosome centromere. The cattle centromere showing methylated cytosines residue frequency at 5kb bins from PacBio and ONT long reads sequencing technology, centromeric protein A (CENP-A) read depth track, centromeric satellite repeats organized into higher-order repeat (HORs) and the percentage identity of the HORs. The centromeric satellite comprised the cattle 73bp monomer (cen-Sat) which were organized into 3.7kb HORs (cen-HOR) in varying tandem configurations of the monomer. The consistently high CpG methylation pattern from the two long read technology across the centromeric satellite array dipped on the flanks of the HOR array. The right flank just after the high identity HOR array coincided with the region of CENP-A enrichment. This is thought to be the locus of the kinetochore assembly on the centromere as previously annotated on human centromeres[5]. (Source data are provided as a Source Data file).

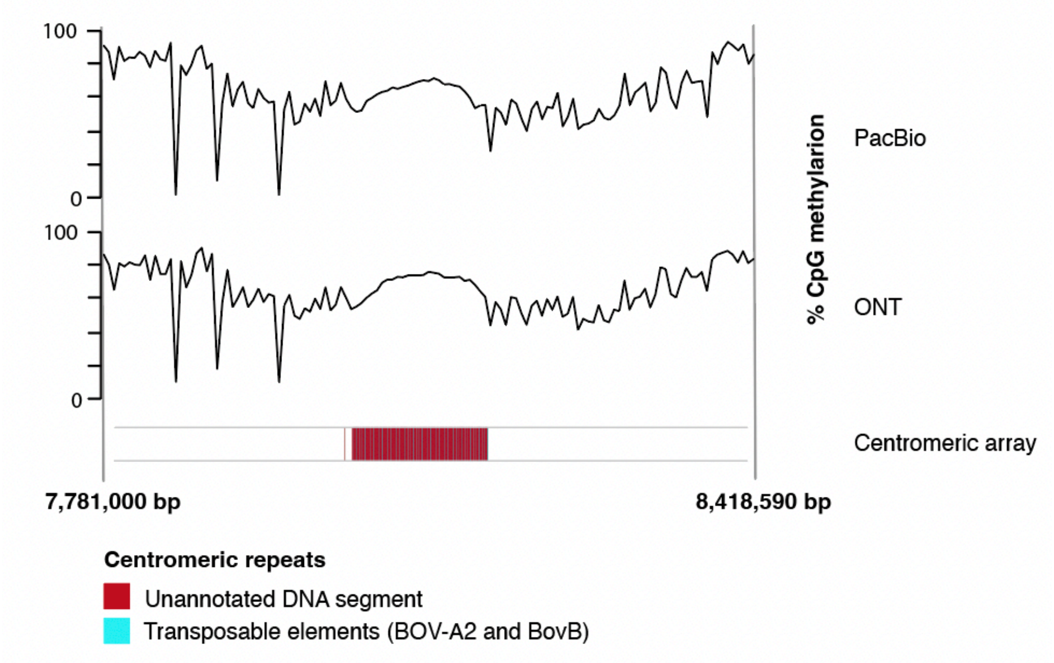

Figure S9: The sheep Y-chromosome centromere. The sheep centromere (8.03-8.15Mb) showing methylated cytosines residue frequency at 5kb bins obtained from PacBio and ONT long reads sequencing technology, and the centromeric array spanning about 112kb. The centromeric array comprised tandem copies of a unit containing the bovine transposable elements *BOV-A2* and *BovB* separated by unannotated DNA. Sharp dips in the consistently high CpG methylation across the centromeric composite array were located at the flanks of the array. (Source data are provided as a Source Data file).

A.

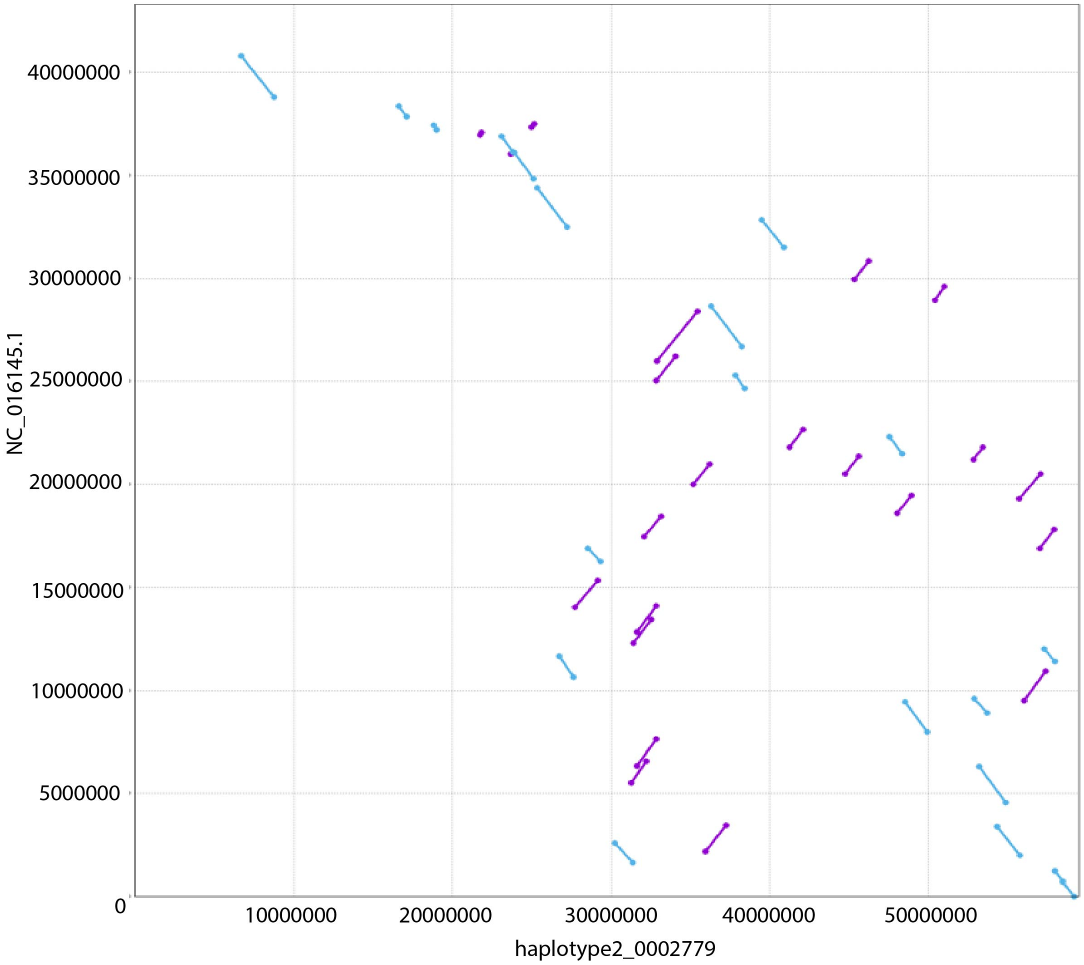

B

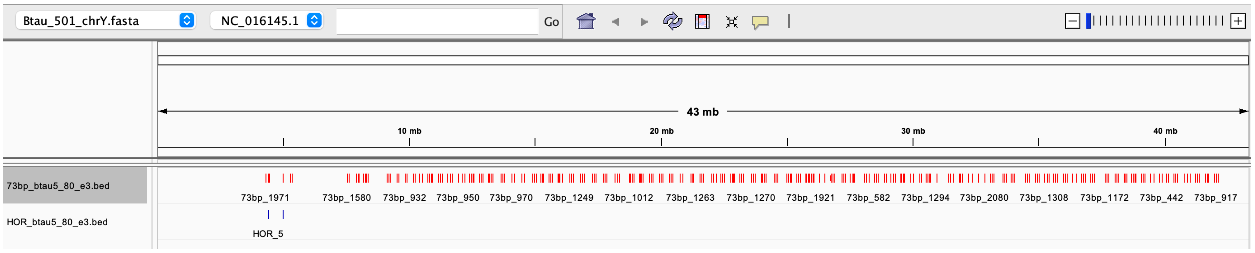

Figure S10: (A) Mashmap[6] alignment between the BTAU5-Y (43.3Mbp) on the Y-axis and the T2T cattle (59.4Mb) chromosomes at 50kb minimum segment length for alignment. Forward orientation alignments are shown in purple color while inverted alignments are shown in light blue color. (B) The cattle 73bp centromeric repeat monomer (red track) and the higher order repeat (HOR) sequence (blue track) on the BTAU5-Y showing just four copies of the HOR around the locus where the centromere is missing from.

A.

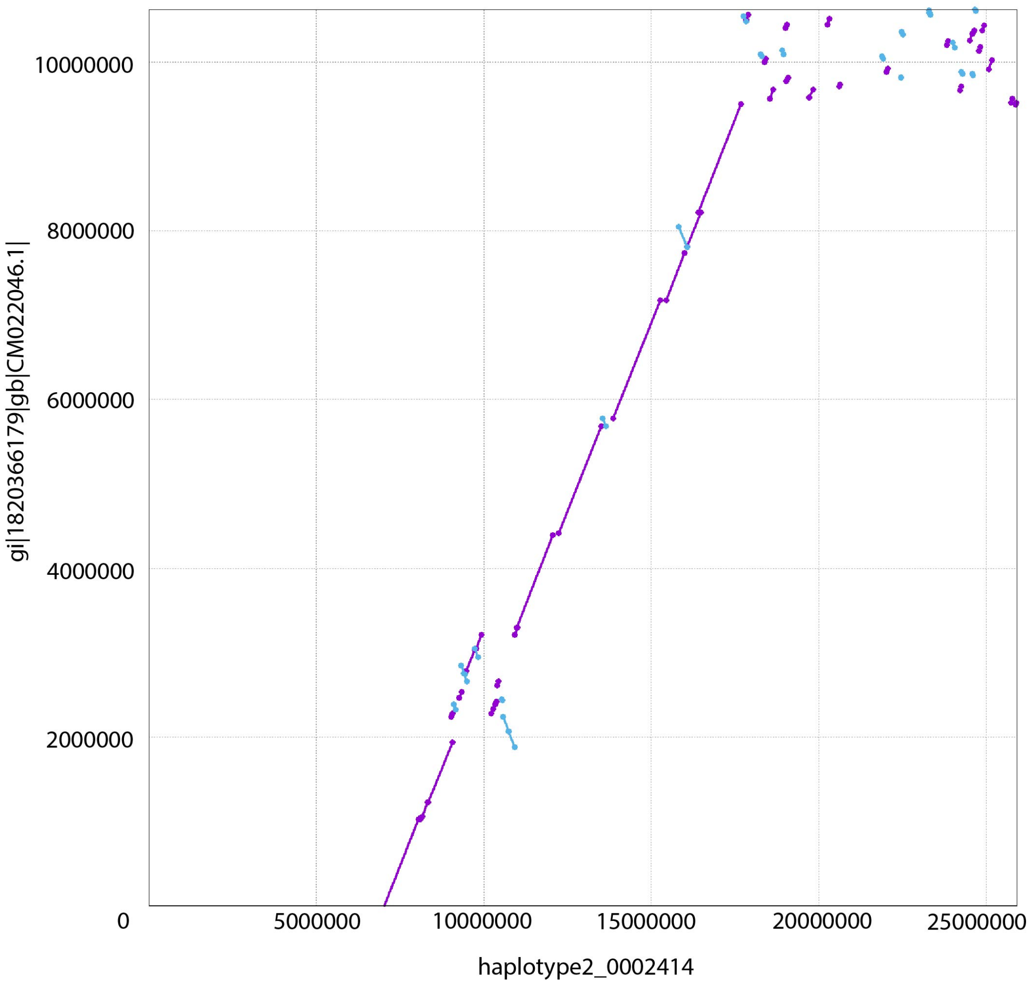

B.

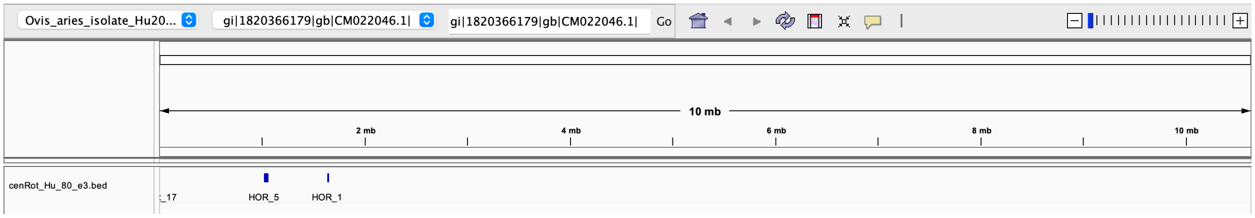

Figure S10: (A) Alignment dot plot between the Hu Sheep (y-axis) and the T2T sheep Y-chromosome (x-axis). Only the MSY was available for the Hu sheep[7], and as such the PAR region spanning 7.01Mb on the T2T sheep (lower left corner) is missing from the alignment. (B) The T2T sheep-derived centromeric repeat on the Hu sheep at 1.02Mb from the beginning of the MSY comprising ten tandemly arrayed copies spanning 35.04kb.

SUPPLEMENTARY METHODS

1. Defining the PAR boundaries

The long reads from a female individual were mapped to the assemblies with minimap2[8] using the following command:

minimap2 -a -t $threads $assembly $reads > $inputTAG".sam"

2. Repeat elements annotation

Repeats element annotation on the Y chromosomes was done with RepeatMasker[9] using the options *-species "bos taurus" -xsmall -no_is -pa $threads -gff.*

3. Satellite DNA annotation with Tandem Repeat Finder (TRF)

Repeats annotation with TRF[10] was run on the two Y-chromosome assemblies using the following parameters:

trf $inFile $match $mismatch $indel $PM $PI $minscore $maxperiod -d -h -ngs > trfOutput.txt

match=2, mismatch=7, indel=7, PM=80, PI=5, minscore=200, maxperiod=2000

4. CENP-A enrichment analysis

Analysis of the CENPA data for peak identification was carried out with SEACR[11] as follows:

bash SEACR_1.3.sh target.bedgraph 0.1 non stringent output

5. Methylated Cytosines analysis (CpG Methylation)

The prescribed PacBio steps for methylated cytosine data analysis

1. Primrose was used to estimate the probability of methylation from the raw reads at each of the CpG sites.
2. The resulting bam files were aligned to the reference assembly with pbmm2 using the command:

```
pbmm2 align \
  -j 48 \
  --sort \
  --log-level INFO \
  --preset HIFI $1 $2 $3, where $1 is the reference assembly, $2 is the file of list
  of modbams, and $3 is the output
```

3. *Aligned_bam_to_cpg_scores* from *pb-CpG-tools* version 2.3.2 was used to produce the percentage of methylated reads across the assembly:

```
aligned_bam_to_cpg_scores \
  --bam $inputBamFile \
  --output-prefix $runPrefix \
  --model pileup_calling_model.v1.tflite \
  --modsites-mode reference \
  --ref $referenceSequence \
```

6. Transcript level quantification on the Y-chromosomes

Following quality control on the RNA-Seq raw reads, the transcripts level was calculated for the Y chromosomes using STAR[12] aligner as follows:

The genome was indexed as follows:

```
STAR --runThreadN $threads --runMode genomeGenerate --genomeDir $pathToGenome \
        --genomeFastaFiles $pathToGenomeFasta --sjdbGTFfile $annotationPath \
        --sjdbGTFtagExonParentTranscript Parent --sjdbOverhang 100
```

The reads were then aligned with STAR in single or paired end reads mode depending on the data.

For single end reads mode:

```
STAR --runThreadN $threads --runMode alignReads --genomeDir $pathToGenome \
        --readFilesIn $pathToReads \
        --readFilesCommand zcat --outFileNamePrefix $pathToOutputFileWithPrefix \
        --outSAMtype BAM SortedByCoordinate --quantMode GeneCounts
```

For paired-end reads mode:

```
STAR --runThreadN $threads --runMode alignReads --genomeDir $pathToGenome \
        --readFilesIn $pathToReads1 $pathToReads2 \
        --readFilesCommand zcat --outFileNamePrefix $pathToOutputFileWithPrefix \
        --outSAMtype BAM SortedByCoordinate --quantMode GeneCounts
```

The output produced was the number of reads aligned to each gene in the supplied annotation file.

REFERENCES

1. Rhie, A., Walenz, B. P., Koren, S. & Phillippy, A. M. Merqury: Reference-free quality, completeness, and phasing assessment for genome assemblies. *Genome Biol* **21**, 1–27 (2020).
2. Wick, R. R., Schultz, M. B., Zobel, J. & Holt, K. E. Bandage: Interactive visualization of de novo genome assemblies. *Bioinformatics* **31**, 3350–3352 (2015).
3. Robinson, J. T. *et al.* Integrative genomics viewer. (2011) doi:10.1038/nbt0111-24.
4. Yang, Y. *et al.* ZNF280BY and ZNF280AY: Autosome derived Y-chromosome gene families in Bovidae. *BMC Genomics* **12**, 9–11 (2011).
5. Gershman, A. *et al.* Epigenetic patterns in a complete human genome. *Science (1979)* **376**, (2022).
6. Jain, C., Koren, S., Dilthey, A., Phillippy, A. M. & Aluru, S. A fast adaptive algorithm for computing whole-genome homology maps. *Bioinformatics* **34**, i748–i756 (2018).
7. Li, R. *et al.* A Hu sheep genome with the first ovine Y chromosome reveal introgression history after sheep domestication. **64**, 1116–1130 (2021).
8. Li, H. Minimap2: Pairwise alignment for nucleotide sequences. *Bioinformatics* **34**, 3094–3100 (2018).
9. Smit AFA, Hubley R & Green P. RepeatMasker Open-4.0. (2013).
10. Benson, G. Tandem repeats finder: a program to analyze DNA sequences. *Nucleic Acids Res* **27**, 573–580 (1999).
11. Meers, M. P., Tenenbaum, D. & Henikoff, S. Peak calling by Sparse Enrichment Analysis for CUT&RUN chromatin profiling. *Epigenetics Chromatin* **12**, (2019).
12. Dobin, A. *et al.* STAR: ultrafast universal RNA-seq aligner. *Bioinformatics* **29**, 15–21 (2013).
