# Supplementary material for: Telomere-to-telomere assemblies of cattle and sheep Y-chromosomes uncover divergent structure and gene content
Source: Nat Commun. 2024 Sep 27;15:8277. doi: 10.1038/s41467-024-52384-5 (PMC11436988; doi:10.1038/s41467-024-52384-5)
Supplement: Supplementary file 3 — Description of Additional Supplementary Files [file 41467_2024_52384_MOESM3_ESM.pdf]

Description of Additional Supplementary Files

File Name: Supplementary Data 1

Description: Statistics of the raw reads from the mix of sequencing technologies used for the cattle and sheep genome assemblies.

File Name: Supplementary Data 2

Description: Genome assembly completeness assessment with lineage-specific orthologous genes using Compleasm. CattleHap2-Wagyu and SheepHap2-Churro are the male haplotypes containing the Y-chromosomes.

File Name: Supplementary Data 3

Description: Repeat content on the cattle and the sheep T2T chromosomes.

File Name: Supplementary Data 4

Description: NCBI annotation of the cattle T2T Y-chromosome.

File Name: Supplementary Data 5

Description: NCBI annotation of the sheep T2T Y-chromosome.

File Name: Supplementary Data 6

Description: Comparison of the protein-coding genes in the PARs on the X- and Y-chromosomes of cattle and sheep.

File Name: Supplementary Data 7

Description: Ampliconic gene families on the cattle and the sheep Y-chromosomes.

File Name: Supplementary Data 8

Description: Cattle Y-chromosome reads count table from RNA-Seq data indicating transcriptional activity.

File Name: Supplementary Data 9

Description: Sheep Y-chromosome reads count table from RNA-Seq data indicating

transcripts activity.

File Name: Supplementary Data 10

Description: Abundance of the cattle-derived 73bp centromeric monomer on the cattle and sheep whole genome assemblies. The sequence appears to be sex chromosome-specific being located on only the sex chromosome contigs of the haplotypes.

File Name: Supplementary Data 11

Description: Centromeric repeat sequence on the cattle and the sheep Y-chromosomes.

File Name: Supplementary Data 12

Description: Mashmap alignment between the BTAU5-Y and the T2T T2T_Cattle Y-chromosome.

File Name: Supplementary Data 13

Description: Comparison of the T2T cattle Y-chromosome to the BTAU5-Y highlighting the missing protein-coding genes in BTAU5-Y relative to the T2T cattle Y-chromosome.

File Name: Supplementary Data 14

Description: Mashmap alignment between the Hu-Sheep MSY and the T2T sheep Y-chromosome.

File Name: Supplementary Data 15

Description: Cattle T2T Y chromosome protein-coding genes annotation with homology search of protein sequences and Liftoff from the X-chromosome.

File Name: Supplementary Data 16

Description: Sheep T2T Y chromosome protein-coding genes annotation with homology search of protein sequences and Liftoff from the X-chromosome.

File Name: Supplementary Data 17

Description: All publicly available cattle assemblies from male individuals on NCBI.

File Name: Supplementary Data 18

Description: All publicly available assemblies from male sheep species on NCBI.
