# Supplementary material for: Telomere-to-telomere assemblies of cattle and sheep Y-chromosomes uncover divergent structure and gene content
Source: Nat Commun. 2024 Sep 27;15:8277. doi: 10.1038/s41467-024-52384-5 (PMC11436988; doi:10.1038/s41467-024-52384-5)
Supplement: Supplementary file 5 — Reporting Summary [file 41467_2024_52384_MOESM5_ESM.pdf]

nature portfolio

Corresponding author(s): Tim P.L. Smith
Brenda M. Murdoch

Last updated by author(s): Aug 7, 2024

Reporting Summary

Nature Portfolio wishes to improve the reproducibility of the work that we publish. This form provides structure for consistency and transparency in reporting. For further information on Nature Portfolio policies, see our Editorial Policies and the Editorial Policy Checklist.

Statistics

For all statistical analyses, confirm that the following items are present in the figure legend, table legend, main text, or Methods section.

n/a	Confirmed	
☐	☒	The exact sample size (*n*) for each experimental group/condition, given as a discrete number and unit of measurement
☐	☒	A statement on whether measurements were taken from distinct samples or whether the same sample was measured repeatedly
☒	☐	The statistical test(s) used AND whether they are one- or two-sided<br>*Only common tests should be described solely by name; describe more complex techniques in the Methods section.*
☒	☐	A description of all covariates tested
☒	☐	A description of any assumptions or corrections, such as tests of normality and adjustment for multiple comparisons
☐	☒	A full description of the statistical parameters including central tendency (e.g. means) or other basic estimates (e.g. regression coefficient) AND variation (e.g. standard deviation) or associated estimates of uncertainty (e.g. confidence intervals)
☒	☐	For null hypothesis testing, the test statistic (e.g. *F*, *t*, *r*) with confidence intervals, effect sizes, degrees of freedom and *P* value noted<br>*Give P values as exact values whenever suitable.*
☒	☐	For Bayesian analysis, information on the choice of priors and Markov chain Monte Carlo settings
☒	☐	For hierarchical and complex designs, identification of the appropriate level for tests and full reporting of outcomes
☒	☐	Estimates of effect sizes (e.g. Cohen's *d*, Pearson's *r*), indicating how they were calculated

Our web collection on statistics for biologists contains articles on many of the points above.

Software and code

Policy information about availability of computer code

Data collection	No software was used for data collection.
Data analysis	Genome assembly was done with Verkko v1.2., Meryl v1.3, and Merqury v1.3 (The complete genome assemblies are still being manually curated prior to later release.)<br>Genome quality assessments were carried out with GfaStats v1.3.6, Merqury v1.3, Compleasm v0.21 and Seqtk 1.4-r130-dirty.<br>Repeats content estimation and identification of the new centromeric satellite repeats were done with RepeatMasker v4.1.2-pl, Tandem Repeat Finder (TRF) v4.09, IGV v2.03, JBrowse v2.3.2, and NCBI/Blast 2.13.0+.<br>Definition of the PAR boundaries and other chromosome features utilized Minimap2 v2.24-r1155-dirty, Mashmap (mashmap-Linux64-v2.0), Bedtools v2.30.0, and IGV v2.15.4.<br>Manual annotation of protein-coding genes was carried out with Liftoff v1.6.3 and Miniprot v0.10-r226-dirty.<br>Transcripts activity analysis was done with FastQC v0.11.9, MultiQC v1.14, Fastp v0.23.4 and STAR v2.7.11a.<br>Phylogenetic analysis of the ampliconic genes families was done with Mafft v7.490 and Unipro Ugene v47.0.<br>CENPA CUT&RUN data was processed with TrimGalore-0.6.10, FastQC v0.12.1, Multiqc v1.15, Picard Tools v3.0.0, Samtools v1.17, Bedtools v2.30.0, Bowtie2 v2.5.2, and SEACR v1.3.<br>CpG methylation calling and analysis were done with Primrose v1.3.0, Dorado v0.3.4, Winnowmap v2.03, Meryl v1.4, pbmm2 v1.9.0, modbam2bed v0.6.3, bedGraphToBigWig v2.10<br>Visualization plots were produced with RStudio v1.4.1717<br>Y chromosome contamination was checked with NCBI FCS adaptor

For manuscripts utilizing custom algorithms or software that are central to the research but not yet described in published literature, software must be made available to editors and reviewers. We strongly encourage code deposition in a community repository (e.g. GitHub). See the Nature Portfolio guidelines for submitting code & software for further information.

Data

Policy information about availability of data

All manuscripts must include a data availability statement. This statement should provide the following information, where applicable:
- Accession codes, unique identifiers, or web links for publicly available datasets
- A description of any restrictions on data availability
- For clinical datasets or third party data, please ensure that the statement adheres to our policy

Source data are provided witht this paper. The T2T Y-chromosomes have been added to the current reference assemblies of cattle and sheep on NCBI. They can also be accessed independently in the NCBI under the accessions GCA_030378505.1 [https://www.ncbi.nlm.nih.gov/datasets/genome/GCA_030378505.1] for the cattle, and GCA_030512445.1 [https://www.ncbi.nlm.nih.gov/datasets/genome/GCA_030512445.1] for the sheep. The methylated cytosines data generated in this study have been deposited in the GEO database under the accession GSE262831 [https://www.ncbi.nlm.nih.gov/geo/query/acc.cgi?acc=GSE262831] for sheep, and GSE263098 [https://www.ncbi.nlm.nih.gov/geo/query/acc.cgi?acc=GSE263098] for cattle. The cattle CENP-A CUT&RUN data generated in this study has been deposited in the GEO database under the accession GSE262830 [https://www.ncbi.nlm.nih.gov/geo/query/acc.cgi?acc=GSE262830]. The RNA-Seq data used for the transcriptional activity analysis in this study are available in NCBI under accession code PRJNA565682 [https://www.ncbi.nlm.nih.gov/nuccore/?term=PRJNA565682] for cattle and PRJNA552574 [https://www.ncbi.nlm.nih.gov/nuccore/?term=PRJNA552574] and PRJNA437085 [https://www.ncbi.nlm.nih.gov/nuccore/?term=PRJNA437085] for sheep.

Research involving human participants, their data, or biological material

Policy information about studies with human participants or human data. See also policy information about sex, gender (identity/presentation), and sexual orientation and race, ethnicity and racism.

Reporting on sex and gender	No human participants were used in this study.
Reporting on race, ethnicity, or other socially relevant groupings	No human participants were used in this study.
Population characteristics	No human participants were used in this study.
Recruitment	No human participants were used in this study.
Ethics oversight	No human participants were used in this study.

Note that full information on the approval of the study protocol must also be provided in the manuscript.

Field-specific reporting

Please select the one below that is the best fit for your research. If you are not sure, read the appropriate sections before making your selection.

[x] Life sciences [ ] Behavioural & social sciences [ ] Ecological, evolutionary & environmental sciences

For a reference copy of the document with all sections, see nature.com/documents/nr-reporting-summary-flat.pdf

Life sciences study design

All studies must disclose on these points even when the disclosure is negative.

Sample size	One sample each of 120-day fetal F1 cross of a Wagyu and a Charolais cattle, and 100-day fetal F1 cross of a Churro and a Friesian sheep were used to generate the complete genome assemblies from which the Y-chromosomes were extracted. A sample is sufficient to generate whole genome sequence of an organism.
Data exclusions	The autosomes and the X-chromosomes of both the cattle and sheep were excluded for this study.
Replication	Replication was carried out on the methylated cytosines data obtained from the same samples using different technologies - PacBio and ONT. The results from the two different sequencing platforms showed very high concordance between them.
Randomization	Randomization is not applicable to this study.
Blinding	Blinding is not applicable to this study.

Reporting for specific materials, systems and methods

We require information from authors about some types of materials, experimental systems and methods used in many studies. Here, indicate whether each material, system or method listed is relevant to your study. If you are not sure if a list item applies to your research, read the appropriate section before selecting a response.

Materials & experimental systems

n/a	Involved in the study
☐	☒ Antibodies
☐	☐ Eukaryotic cell lines
☐	☐ Palaeontology and archaeology
☐	☒ Animals and other organisms
☐	☐ Clinical data
☐	☐ Dual use research of concern
☐	☐ Plants

Methods

n/a	Involved in the study
☐	☒ ChIP-seq
☐	☐ Flow cytometry
☐	☐ MRI-based neuroimaging

Antibodies

Antibodies used	The GeneTex CENP-A antibody with catalog number GTX13939 was used for the CENP-A pull down assay in this study. The antibody is monoclonal and of the isotype IgG1. Although it is stated on the manufacturer's website that the antibody reacts with Human, Chicken, and Caenorhabditis elegans, there is also evidence of reactivity with calf and goat on the datasheet provided by the manufacturer at https://www.genetex.com/PDF/Download?catno=GTX13939.
Validation	The antibody has been sufficiently validated in multiple studies reported on the manufacturer's website such as: Saayman et al. (2023), Wu et al. (2023), Pesenti et al. (2022), among others.

Eukaryotic cell lines

Policy information about cell lines and Sex and Gender in Research

Cell line source(s)	*State the source of each cell line used and the sex of all primary cell lines and cells derived from human participants or vertebrate models.*
Authentication	*Describe the authentication procedures for each cell line used OR declare that none of the cell lines used were authenticated.*
Mycoplasma contamination	*Confirm that all cell lines tested negative for mycoplasma contamination OR describe the results of the testing for mycoplasma contamination OR declare that the cell lines were not tested for mycoplasma contamination.*
Commonly misidentified lines (See ICLAC register)	*Name any commonly misidentified cell lines used in the study and provide a rationale for their use.*

Palaeontology and Archaeology

Specimen provenance	*Provide provenance information for specimens and describe permits that were obtained for the work (including the name of the issuing authority, the date of issue, and any identifying information). Permits should encompass collection and, where applicable, export.*
Specimen deposition	*Indicate where the specimens have been deposited to permit free access by other researchers.*
Dating methods	*If new dates are provided, describe how they were obtained (e.g. collection, storage, sample pretreatment and measurement), where they were obtained (i.e. lab name), the calibration program and the protocol for quality assurance OR state that no new dates are provided.*

☐ Tick this box to confirm that the raw and calibrated dates are available in the paper or in Supplementary Information.

Ethics oversight	*Identify the organization(s) that approved or provided guidance on the study protocol, OR state that no ethical approval or guidance was required and explain why not.*

Note that full information on the approval of the study protocol must also be provided in the manuscript.

Animals and other research organisms

Policy information about studies involving animals; ARRIVE guidelines recommended for reporting animal research, and Sex and Gender in Research

Laboratory animals	This study did not involve laboratory animals.
Wild animals	This study did not involve wild animals.
Reporting on sex	Y-chromosomes, being the sex-determining chromosome and present only in male individuals were obtained from cattle and sheep male animals. The results in this study are thus specific to male animals.

Field-collected samples	This does not apply to this study.
Ethics oversight	This study was carried out in accordance with the University of Idaho Institutional Animal Care and Use Committee (IACUC) approved protocol with number IACUC-2020-58 for sheep and IACUC-2021-21 for cattle. The male Angus calf which produced the bovine satellite cells used for the CENP-A pull down assay was harvested under the IACUC #017090 also at the University of Idaho.

Note that full information on the approval of the study protocol must also be provided in the manuscript.

Clinical data

Policy information about clinical studies

All manuscripts should comply with the ICMJEguidelines for publication of clinical research and a completedCONSORT checklist must be included with all submissions.

Clinical trial registration	*Provide the trial registration number from ClinicalTrials.gov or an equivalent agency.*
Study protocol	*Note where the full trial protocol can be accessed OR if not available, explain why.*
Data collection	*Describe the settings and locales of data collection, noting the time periods of recruitment and data collection.*
Outcomes	*Describe how you pre-defined primary and secondary outcome measures and how you assessed these measures.*

Dual use research of concern

Policy information about dual use research of concern

Hazards

Could the accidental, deliberate or reckless misuse of agents or technologies generated in the work, or the application of information presented in the manuscript, pose a threat to:

No	Yes	
☒	☐	Public health
☒	☐	National security
☒	☐	Crops and/or livestock
☒	☐	Ecosystems
☒	☐	Any other significant area

Experiments of concern

Does the work involve any of these experiments of concern:

No	Yes	
☒	☐	Demonstrate how to render a vaccine ineffective
☒	☐	Confer resistance to therapeutically useful antibiotics or antiviral agents
☒	☐	Enhance the virulence of a pathogen or render a nonpathogen virulent
☒	☐	Increase transmissibility of a pathogen
☒	☐	Alter the host range of a pathogen
☒	☐	Enable evasion of diagnostic/detection modalities
☒	☐	Enable the weaponization of a biological agent or toxin
☒	☐	Any other potentially harmful combination of experiments and agents

Plants

Seed stocks

Report on the source of all seed stocks or other plant material used. If applicable, state the seed stock centre and catalogue number. If plant specimens were collected from the field, describe the collection location, date and sampling procedures.

Novel plant genotypes

Describe the methods by which all novel plant genotypes were produced. This includes those generated by transgenic approaches, gene editing, chemical/radiation-based mutagenesis and hybridization. For transgenic lines, describe the transformation method, the number of independent lines analyzed and the generation upon which experiments were performed. For gene-edited lines, describe the editor used, the endogenous sequence targeted for editing, the targeting guide RNA sequence (if applicable) and how the editor was applied.

Authentication

Describe any authentication procedures for each seed stock used or novel genotype generated. Describe any experiments used to assess the effect of a mutation and, where applicable, how potential secondary effects (e.g. second site T-DNA insertions, mosiacism, off-target gene editing) were examined.

ChIP-seq

Data deposition

☒ Confirm that both raw and final processed data have been deposited in a public database such as GEO.

☐ Confirm that you have deposited or provided access to graph files (e.g. BED files) for the called peaks.

Data access links
May remain private before publication.

For "Initial submission" or "Revised version" documents, provide reviewer access links. For your "Final submission" document, provide a link to the deposited data.

Files in database submission

Provide a list of all files available in the database submission.

Genome browser session
(e.g. UCSC)

Provide a link to an anonymized genome browser session for "Initial submission" and "Revised version" documents only, to enable peer review. Write "no longer applicable" for "Final submission" documents.

Methodology

Replicates

Replication does not apply to this study.

Sequencing depth

The Cleavage Under Targets & Release Using Nuclease (CUT&RUN) protocol was employed for the CENP-A pull down assay followed by sequencing.
The libraries are paired end for the sample and the input. The reads statistics are as follows:
LIB204270 - Sample
75,093,682 - total reads passed QC
62,073,989 - mapped reads

LIB204271 - Input
75,624,530 - total reads passed QC
39,554,675 - mapped reads

Antibodies

The GeneTex CENP-A antibody with catalog number GTX13939 was used for the CENP-A pull down assay in this study. The antibody is monoclonal and of the isotype IgG1. Although it is stated on the manufacturer's website that the antibody reacts with Human, Chicken, and Caenorhabditis elegans, there is also evidence of reactivity with calf and goat on the datasheet provided by the manufacturer at https://www.genetex.com/PDF/Download?catno=GTX13939.

Peak calling parameters

Alignment with bowtie2 and post-processing
bowtie2 -p96 -k 100 -x {Cattle_reference_assembly_on_NCBI} -1 {sampleRead1} -2 {sampleRead2} | samtools sort -o {sample.sorted.bam}
java -jar picard.jar MarkDuplicates I={sample.sorted.bam} O={sample.sorted_no_dups.bam} M={metrics.log} REMOVE_DUPLICATES=true
samtools view -b -q 20 {sample.sorted_no_dups.bam} -o {sample.sorted_no_dups_20.bam}
samtools sort -n {sample.sorted_no_dups_20.bam} -o {sample.sorted_no_dups_20_name_sorted.bam}
bedtools bamtobed -bedpe -i {sample.sorted_no_dups_20_name_sorted.bam} > {sample.sorted_no_dups_20_name_sorted.bed}
awk '$1==$4 && $6-$2 < 1000 {print $0}' {sample.sorted_no_dups_20_name_sorted.bed} | cut -f 1,2,6 | sort -k1,1 -k2,2n -k3,3n > {sample.sorted_no_dups_20_name_sorted.clean.fragments.bed}
grep -vE '^\.' {sample.sorted_no_dups_20_name_sorted.clean.fragments.bed} > {sample.sorted_no_dups_20_name_sorted.clean.fragments.2.bed}
bedtools genomecov -bg -i {sample.sorted_no_dups_20_name_sorted.clean.fragments.2.bed} -g {Cattle_reference_assembly_on_NCBI.genome} > {sample.sorted_no_dups_20_name_sorted.clean.fragments.2.bedgraph}
SEACR peaks call
SEACR_1.3.sh {sample.sorted_no_dups_20_name_sorted.clean.fragments.2.bedgraph} 0.01 non stringent {calledPeaks}

Data quality

Sequence libraries were checked for quality using FastqQC and adapters were removed before further processing.

Software

SEACR v1.3 was used to call peaks from the CENP-A CUT&RUN data. The tool is a shell script that has options to call peaks either with

or without an input. SEACR takes its input files in the bedgraph format. In the stringent mode which was used in our analysis, enriched regions in target data are called by selecting the top 1% of regions by AUC.
SEACR was run with the command:
SEACR_1.3.sh {sample.bedgraph} 0.01 non stringent {calledPeaks.bed}

Flow Cytometry

Plots

Confirm that:

- [ ] The axis labels state the marker and fluorochrome used (e.g. CD4-FITC).
- [ ] The axis scales are clearly visible. Include numbers along axes only for bottom left plot of group (a 'group' is an analysis of identical markers).
- [ ] All plots are contour plots with outliers or pseudocolor plots.
- [ ] A numerical value for number of cells or percentage (with statistics) is provided.

Methodology

Sample preparation	*Describe the sample preparation, detailing the biological source of the cells and any tissue processing steps used.*
Instrument	*Identify the instrument used for data collection, specifying make and model number.*
Software	*Describe the software used to collect and analyze the flow cytometry data. For custom code that has been deposited into a community repository, provide accession details.*
Cell population abundance	*Describe the abundance of the relevant cell populations within post-sort fractions, providing details on the purity of the samples and how it was determined.*
Gating strategy	*Describe the gating strategy used for all relevant experiments, specifying the preliminary FSC/SSC gates of the starting cell population, indicating where boundaries between "positive" and "negative" staining cell populations are defined.*

- [ ] Tick this box to confirm that a figure exemplifying the gating strategy is provided in the Supplementary Information.

Magnetic resonance imaging

Experimental design

Design type	*Indicate task or resting state; event-related or block design.*
Design specifications	*Specify the number of blocks, trials or experimental units per session and/or subject, and specify the length of each trial or block (if trials are blocked) and interval between trials.*
Behavioral performance measures	*State number and/or type of variables recorded (e.g. correct button press, response time) and what statistics were used to establish that the subjects were performing the task as expected (e.g. mean, range, and/or standard deviation across subjects).*

Acquisition

Imaging type(s)	*Specify: functional, structural, diffusion, perfusion.*
Field strength	*Specify in Tesla*
Sequence & imaging parameters	*Specify the pulse sequence type (gradient echo, spin echo, etc.), imaging type (EPI, spiral, etc.), field of view, matrix size, slice thickness, orientation and TE/TR/flip angle.*
Area of acquisition	*State whether a whole brain scan was used OR define the area of acquisition, describing how the region was determined.*

Diffusion MRI   [ ] Used   [x] Not used

Preprocessing

Preprocessing software	*Provide detail on software version and revision number and on specific parameters (model/functions, brain extraction, segmentation, smoothing kernel size, etc.).*
Normalization	*If data were normalized/standardized, describe the approach(es): specify linear or non-linear and define image types used for transformation OR indicate that data were not normalized and explain rationale for lack of normalization.*

Normalization template | *Describe the template used for normalization/transformation, specifying subject space or group standardized space (e.g. original Talairach, MNI305, ICBM152) OR indicate that the data were not normalized.*

Noise and artifact removal | *Describe your procedure(s) for artifact and structured noise removal, specifying motion parameters, tissue signals and physiological signals (heart rate, respiration).*

Volume censoring | *Define your software and/or method and criteria for volume censoring, and state the extent of such censoring.*

Statistical modeling & inference

Model type and settings | *Specify type (mass univariate, multivariate, RSA, predictive, etc.) and describe essential details of the model at the first and second levels (e.g. fixed, random or mixed effects; drift or auto-correlation).*

Effect(s) tested | *Define precise effect in terms of the task or stimulus conditions instead of psychological concepts and indicate whether ANOVA or factorial designs were used.*

Specify type of analysis: ☐ Whole brain ☐ ROI-based ☐ Both

Statistic type for inference (See Eklund et al. 2016) | *Specify voxel-wise or cluster-wise and report all relevant parameters for cluster-wise methods.*

Correction | *Describe the type of correction and how it is obtained for multiple comparisons (e.g. FWE, FDR, permutation or Monte Carlo).*

Models & analysis

n/a	Involved in the study
☒	☐ Functional and/or effective connectivity
☒	☐ Graph analysis
☒	☐ Multivariate modeling or predictive analysis
